# Crystal structure of fipronil

**DOI:** 10.1107/S205698901701310X

**Published:** 2017-09-15

**Authors:** Hyunjin Park, Jineun Kim, Eunjin Kwon, Tae Ho Kim

**Affiliations:** a Department of Chemistry (BK21 plus) and Research Institute of Natural Sciences, Gyeongsang National University, Jinju 52828, Republic of Korea

**Keywords:** crystal structure, fipronil, acaricides, insecticides

## Abstract

The title compound, C_12_H_4_Cl_2_F_6_N_4_OS {systematic name: 5-amino-1-[2,6-di­chloro-4-(tri­fluoro­meth­yl)phen­yl]-4-[(tri­fluoro­methane)sulfinyl]-1*H*-pyrazole-3-carbo­nitrile}, is a member of the phenyl­pyrazole group of acaricides, and one of the phenyl­pyrazole group of insecticides. The dihedral angle between the planes of the pyrazole and benzene rings is 89.03 (9)°. The fluorine atoms of the tri­fluoro­methyl substituent on the benzene ring are disordered over two sets of sites, with occupancy ratios 0.620 (15):0.380 (15). In the crystal, C—N⋯π inter­actions [N⋯ring centroid = 3.607 (4) Å] together with N—H⋯N and C—H⋯F hydrogen bonds form a looped chain structure along [10]. Finally, N—H⋯O hydrogen bonds and C—Cl⋯π inter­actions [Cl⋯ring centroid = 3.5159 (16) Å] generate a three-dimensional structure. Additionally, there are a short inter­molecular F⋯ F contacts present.

## Abstract 2

The mol­ecular and crystal structure of the insecticide fipronil is reported. In the crystal, N—H⋯N, N—H⋯O, C—H⋯F hydrogen bonds, C—N⋯π and C—Cl⋯π inter­actions link adjacent mol­ecules, forming a three-dimensional network. In addition, there are short F⋯F inter­actions present.

## Chemical context   

Fipronil is an insecticide that belongs to the phenyl­pyrazole group. It is an insecticide with extended use in the control of many agricultural vermin. Fipronil contains a tri­fluoro­methyl­sulfinyl substituent that is not present in any other agrochemicals and this is thought to contribute to its remarkable potency in the field (Hainzl & Casida, 1996 ▸). In addition, it is a highly effective and broad-spectrum insecticide against piercing–sucking, contact and chewing pests and is widely used to control many species of soil and foliar insects on various crops including rice, vegetables and fruits (Kaur *et al.*, 2015 ▸). The toxicity of fipronil is attributed to its ability to act at the GABA receptor as a non-competitive inhibitor of the GABA-gated chloride channels of neurons in the central nervous system. Impediments to the influx of the chloride ions affect the transmission of nervous impulses, causing insect death by neuronal hyperexcitation and paralysis (Medeiros *et al.*, 2015 ▸). Recently, eggs contaminated with fipronil have been found in Europe, Hong Kong and the Republic of Korea. We report here the crystal structure of fipronil, 5-amino-1-[2,6-di­chloro-4-(tri­fluoro­meth­yl)phen­yl]-4-(tri­fluoro­methane­sul­fin­yl)-1*H*-pyrazole-3-carbo­nitrile.
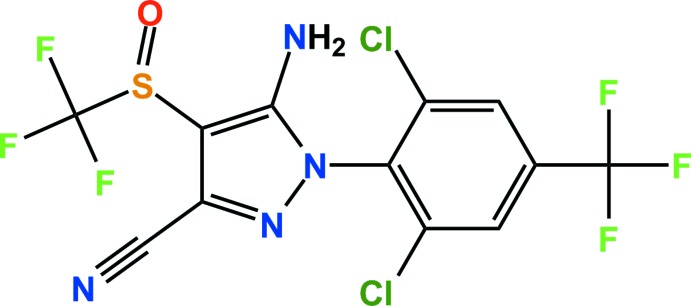


## Structural commentary   

**Figure 1:**
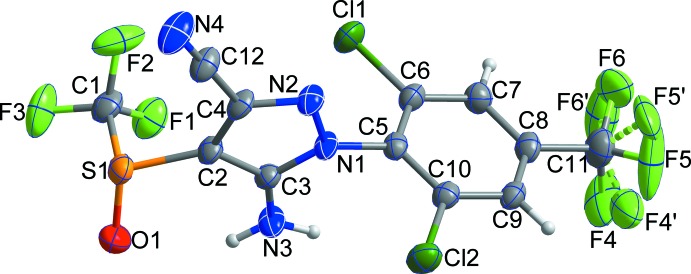
The structure of the title compound, with displacement ellipsoids drawn at the 50% probability level. H atoms are shown as small spheres of arbitrary radius.

The mol­ecular structure of the title compound is shown in Fig. 1 ▸. The dihedral angle between the planes of the pyrazole and benzene rings is 89.03 (9)°. All bond lengths and bond angles are normal and comparable to those observed in similar crystal structures (Kang *et al.*, 2015 ▸; Jiang & Xu, 2009 ▸).

## Supra­molecular features   

**Figure 2:**
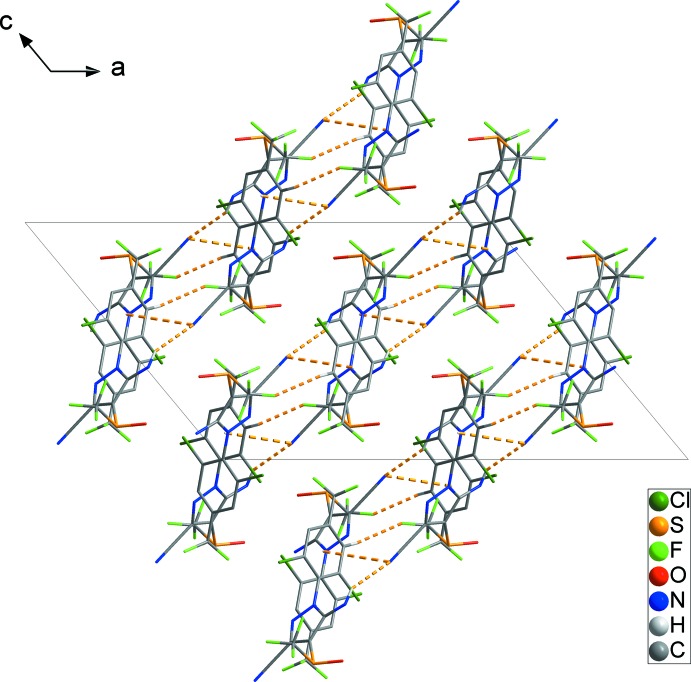
A view along the *b* axis of the crystal packing of the title compound. Looped chains are formed through inter­molecular C—N⋯π inter­actions together with N—H⋯N and C—H⋯F hydrogen bonds (yellow dashed lines). H atoms not involved in inter­molecular inter­actions have been omitted for clarity.

**Figure 3:**
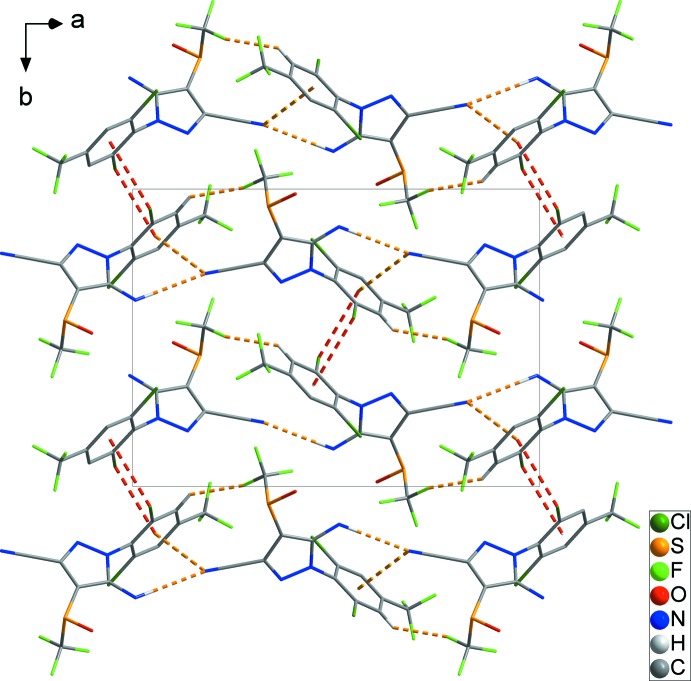
The two-dimensional network formed through inter­molecular C—Cl⋯π inter­actions (red dashed lines). H atoms not involved in inter­molecular inter­actions have been omitted for clarity.

**Figure 4:**
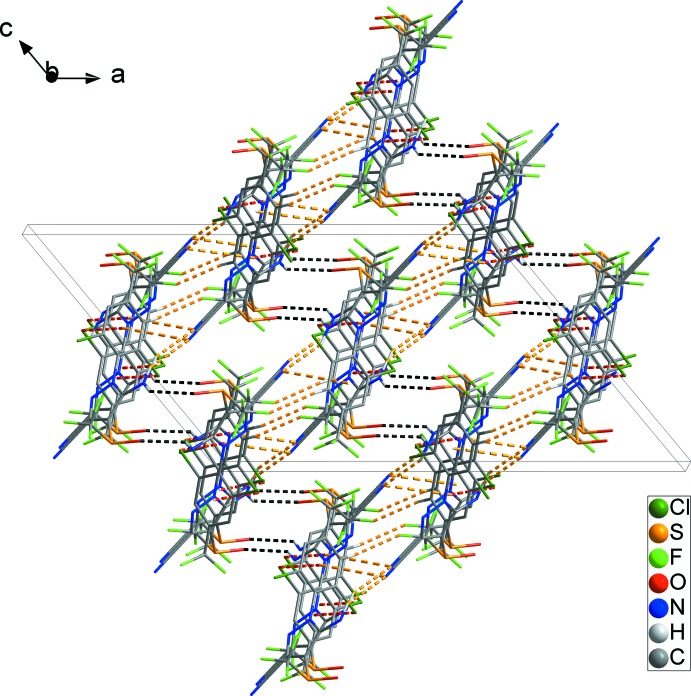
The overall packing of the title compound, showing the three-dimensional network formed through N—H⋯O hydrogen bonds (black dashed lines). H atoms not involved in inter­molecular inter­actions have been omitted for clarity.

In the crystal, mol­ecules are linked by C12—N4⋯*Cg*1^iii^ inter­actions [N⋯*Cg*1 = 3.607 (4) Å; *Cg*1 is the centroid of the C5–C10 ring; symmetry code: (iii) −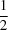 + *x*, 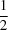 − *y*, −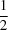 + *z*], together with N3—H3*A*⋯N4^i^ and C9—H9⋯F2^i^ hydrogen bonds, forming looped chains along [10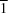] (Fig. 2 ▸). Inversion-related C10—Cl2⋯*Cg*1^iv^ inter­actions [Cl⋯*Cg*1 = 3.5159 (16)Å; symmetry code: (iv) 2 − *x*, −*y*, 2 − *z*] (red dashed lines), link adjacent chains, resulting in a two-dimensional network parallel to the (10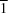) plane (Fig. 3 ▸). Finally, classical N3—H3*B*⋯O1^ii^ hydrogen bonds (black dashed lines) combine with these contacts to generate a three-dimensional network structure (Fig. 4 ▸ and Table 1 ▸). Short F2⋯F4′^v^ [2.762 (14) Å] and F3⋯F6′^vi^ [2.855 (12) Å] inter­actions are also present [symmetry codes: (v) −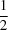 + *x*, 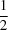 − *y*, −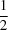 + *z*; (vi) −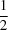 + *x*, 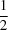 + *y*, −1 + *z*].

## Database survey   

The title compound has been used as a starting material for the synthesis of other materials (Tang *et al.*, 2005 ▸; Liu *et al.*, 2013 ▸). Moreover, the structures of Cu^II^, Cd^II^, Zn^II^ and Mn^II^ complexes using fipronil as a ligand are known (Tang *et al.*, 2009 ▸, 2010 ▸). The crystal structures of other phenyl­pyrazole compounds such as ethyl 7-methyl-2-phenyl­pyrazolo­[1,5-*a*]pyrimidine-5-carboxyl­ate (Bassoude *et al.*, 2013 ▸) and 4-{[(*E*)-(3,5-dimethyl-1-phenyl-1*H-*pyrazol-4-yl)methyl­idene]amino}-1,5-dimethyl-2-phenyl-1*H*-pyrazol-3(2*H*)-one (Fun *et al.*, 2010 ▸) have also been reported.

## Synthesis and crystallization   

The title compound was purchased from Dr. Ehrenstorfer GmbH. Colourless single crystals suitable for X-ray diffraction were obtained from a CH_3_CN solution by slow evaporation at room temperature.

## Refinement   

Crystal data, data collection and structure refinement details are summarized in Table 2 ▸. All H atoms were positioned geometrically and refined using a riding model with *d*(N—H) = 0.88 Å, *U*_iso_ = 1.2*U*_eq_(C) for the N—H group, *d*(C—H) = 0.95 Å, *U*_iso_ = 1.2*U*_eq_(C) for aromatic C—H. Atoms F4–F6 of the CF_3_ substituent are disordered over two sets of sites. Their occupancies refined to 0.620 (15) and 0.380 (15).

## Supplementary Material

Crystal structure: contains datablock(s) I, New_Global_Publ_Block. DOI: 10.1107/S205698901701310X/sj5534sup1.cif

Structure factors: contains datablock(s) I. DOI: 10.1107/S205698901701310X/sj5534Isup2.hkl

CCDC reference: 1574261

Additional supporting information:  crystallographic information; 3D view; checkCIF report

## Figures and Tables

**Table 1:** Hydrogen-bond geometry (Å, °)

*D*—H⋯*A*	*D*—H	H⋯*A*	*D*⋯*A*	*D*—H⋯*A*
N3—H3*A*⋯N4^i^	0.88	2.39	3.183 (3)	151
N3—H3*B*⋯O1^ii^	0.88	2.28	2.896 (3)	127
C9—H9⋯F2^i^	0.95	2.42	3.222 (3)	143

Symmetry codes: (i) 
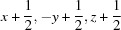
; (ii) 
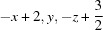
.

**Table 2:** Experimental details

Crystal data	
Chemical formula	C_12_H_4_Cl_2_F_6_N_4_OS
*M*_r_	437.15
Crystal system, space group	Monoclinic, *C*2/*c*
Temperature (K)	173
*a*, *b*, *c* (Å)	22.5649 (16), 12.6823 (9), 14.9051 (11)
β (°)	129.699 (3)
*V* (Å^3^)	3281.9 (4)
*Z*	8
Radiation type	Mo *K*α
μ (mm^−1^)	0.60
Crystal size (mm)	0.15 × 0.13 × 0.04

Data collection	
Diffractometer	Bruker APEXII CCD
Absorption correction	Multi-scan (*SADABS*; Bruker, 2014 ▸)
*T*_min_, *T*_max_	0.587, 0.746
No. of measured, independent and observed [*I* > 2σ(*I*)] reflections	14125, 3731, 2506
*R*_int_	0.066
(sin θ/λ)_max_ (Å^−1^)	0.648

Refinement	
*R*[*F* ^2^ > 2σ(*F* ^2^)], *wR*(*F* ^2^), *S*	0.048, 0.120, 1.03
No. of reflections	3731
No. of parameters	263
No. of restraints	36
H-atom treatment	H-atom parameters constrained
Δρ_max_, Δρ_min_ (e Å^−3^)	0.38, −0.32

Computer programs: *APEX2* and *SAINT* (Bruker, 2014 ▸), *SHELXS97* and *SHELXTL* (Sheldrick, 2008 ▸), *SHELXL2014* (Sheldrick, 2015 ▸), *DIAMOND* (Brandenburg, 2010 ▸) and *publCIF* (Westrip, 2010 ▸).

## supplementary crystallographic information

### Crystal data

**Table:**

C_12_H_4_Cl_2_F_6_N_4_OS	*F*(000) = 1728
*M**_r_* = 437.15	*D*_x_ = 1.769 Mg m^−^^3^
Monoclinic, *C*2/*c*	Mo *K*α radiation, λ = 0.71073 Å
*a* = 22.5649 (16) Å	Cell parameters from 2101 reflections
*b* = 12.6823 (9) Å	θ = 2.4–21.6°
*c* = 14.9051 (11) Å	µ = 0.60 mm^−^^1^
β = 129.699 (3)°	*T* = 173 K
*V* = 3281.9 (4) Å^3^	Plate, colourless
*Z* = 8	0.15 × 0.13 × 0.04 mm

### Data collection

**Table:**

Bruker APEXII CCD diffractometer	2506 reflections with *I* > 2σ(*I*)
φ and ω scans	*R*_int_ = 0.066
Absorption correction: multi-scan (SADABS; Bruker, 2014)	θ_max_ = 27.4°, θ_min_ = 2.0°
*T*_min_ = 0.587, *T*_max_ = 0.746	*h* = −28→29
14125 measured reflections	*k* = −16→16
3731 independent reflections	*l* = −19→19

### Refinement

**Table:**

Refinement on *F*^2^	36 restraints
Least-squares matrix: full	Hydrogen site location: inferred from neighbouring sites
*R*[*F*^2^ > 2σ(*F*^2^)] = 0.048	H-atom parameters constrained
*wR*(*F*^2^) = 0.120	*w* = 1/[σ^2^(*F*_o_^2^) + (0.0463*P*)^2^ + 0.8977*P*] where *P* = (*F*_o_^2^ + 2*F*_c_^2^)/3
*S* = 1.03	(Δ/σ)_max_ = 0.001
3731 reflections	Δρ_max_ = 0.38 e Å^−^^3^
263 parameters	Δρ_min_ = −0.32 e Å^−^^3^

### Special details

**Table:**

Geometry. All esds (except the esd in the dihedral angle between two l.s. planes) are estimated using the full covariance matrix. The cell esds are taken into account individually in the estimation of esds in distances, angles and torsion angles; correlations between esds in cell parameters are only used when they are defined by crystal symmetry. An approximate (isotropic) treatment of cell esds is used for estimating esds involving l.s. planes.

### Fractional atomic coordinates and isotropic or equivalent isotropic displacement parameters (Å^2^)

**Table:**

	*x*	*y*	*z*	*U*_iso_*/*U*_eq_	Occ. (<1)
Cl1	0.94088 (4)	0.32995 (6)	1.06637 (7)	0.0429 (2)	
Cl2	1.04310 (4)	0.06765 (6)	0.90739 (7)	0.0417 (2)	
S1	0.83382 (4)	0.44166 (6)	0.63886 (6)	0.0337 (2)	
F1	0.89072 (10)	0.55280 (14)	0.83001 (16)	0.0516 (5)	
F2	0.77293 (11)	0.50457 (17)	0.7312 (2)	0.0695 (7)	
F3	0.80245 (12)	0.62984 (14)	0.66925 (18)	0.0673 (6)	
F4	1.2426 (3)	0.1359 (8)	1.3877 (5)	0.084 (2)	0.620 (15)
F5	1.1906 (7)	−0.0112 (5)	1.3561 (5)	0.110 (4)	0.620 (15)
F6	1.1641 (4)	0.1123 (8)	1.4199 (6)	0.075 (2)	0.620 (15)
F4'	1.2399 (5)	0.0680 (16)	1.3697 (9)	0.081 (4)	0.380 (15)
F5'	1.1577 (6)	−0.0083 (10)	1.3614 (9)	0.075 (3)	0.380 (15)
F6'	1.1875 (10)	0.1483 (11)	1.4220 (10)	0.092 (5)	0.380 (15)
O1	0.89951 (12)	0.48383 (17)	0.6504 (2)	0.0470 (6)	
N1	0.93791 (12)	0.22985 (17)	0.88474 (19)	0.0279 (5)	
N2	0.86486 (12)	0.18765 (17)	0.8196 (2)	0.0321 (6)	
N3	1.00700 (13)	0.37326 (19)	0.8898 (2)	0.0376 (6)	
H3A	1.0499	0.3510	0.9563	0.045*	
H3B	1.0072	0.4310	0.8572	0.045*	
N4	0.67806 (14)	0.2159 (2)	0.5679 (3)	0.0525 (8)	
C1	0.82489 (17)	0.5375 (2)	0.7227 (3)	0.0398 (8)	
C2	0.86620 (15)	0.3372 (2)	0.7357 (2)	0.0291 (6)	
C3	0.94140 (15)	0.3194 (2)	0.8382 (2)	0.0278 (6)	
C4	0.82316 (15)	0.2528 (2)	0.7293 (2)	0.0304 (6)	
C5	0.99688 (14)	0.1918 (2)	0.9991 (2)	0.0268 (6)	
C6	1.00414 (16)	0.2340 (2)	1.0918 (3)	0.0302 (6)	
C7	1.06225 (16)	0.2006 (2)	1.2042 (2)	0.0325 (7)	
H7	1.0673	0.2297	1.2676	0.039*	
C8	1.11308 (15)	0.1241 (2)	1.2236 (2)	0.0319 (7)	
C9	1.10661 (15)	0.0802 (2)	1.1335 (2)	0.0307 (7)	
H9	1.1411	0.0264	1.1480	0.037*	
C10	1.04895 (15)	0.1158 (2)	1.0209 (2)	0.0287 (6)	
C11	1.1763 (2)	0.0878 (3)	1.3460 (3)	0.0481 (9)	
C12	0.74238 (17)	0.2320 (2)	0.6396 (3)	0.0364 (7)	

### Atomic displacement parameters (Å^2^)

**Table:**

	*U*^11^	*U*^22^	*U*^33^	*U*^12^	*U*^13^	*U*^23^
Cl1	0.0409 (5)	0.0398 (4)	0.0512 (5)	0.0144 (3)	0.0308 (4)	0.0070 (3)
Cl2	0.0381 (4)	0.0498 (5)	0.0383 (5)	0.0030 (3)	0.0249 (4)	−0.0050 (3)
S1	0.0242 (4)	0.0383 (4)	0.0292 (4)	0.0042 (3)	0.0127 (4)	0.0077 (3)
F1	0.0376 (11)	0.0530 (12)	0.0410 (12)	0.0005 (9)	0.0143 (10)	−0.0068 (8)
F2	0.0514 (13)	0.0723 (15)	0.1067 (19)	−0.0067 (11)	0.0606 (14)	−0.0199 (13)
F3	0.0577 (13)	0.0370 (11)	0.0654 (14)	0.0157 (9)	0.0200 (12)	0.0090 (9)
F4	0.026 (2)	0.100 (5)	0.060 (3)	−0.007 (3)	−0.003 (2)	0.023 (3)
F5	0.138 (7)	0.048 (3)	0.041 (3)	0.043 (4)	0.010 (3)	0.010 (2)
F6	0.059 (3)	0.125 (6)	0.037 (3)	0.020 (3)	0.030 (3)	0.022 (3)
F4'	0.039 (4)	0.128 (9)	0.063 (5)	0.019 (5)	0.026 (4)	0.048 (5)
F5'	0.062 (5)	0.087 (6)	0.058 (4)	0.006 (4)	0.031 (4)	0.046 (4)
F6'	0.103 (8)	0.081 (6)	0.030 (4)	0.037 (6)	0.013 (5)	−0.001 (4)
O1	0.0429 (13)	0.0494 (14)	0.0594 (15)	0.0056 (11)	0.0376 (13)	0.0151 (11)
N1	0.0143 (11)	0.0307 (12)	0.0257 (13)	−0.0012 (9)	0.0067 (11)	0.0049 (9)
N2	0.0178 (11)	0.0313 (13)	0.0332 (14)	−0.0032 (10)	0.0098 (11)	0.0022 (10)
N3	0.0169 (12)	0.0442 (15)	0.0356 (15)	−0.0038 (10)	0.0093 (12)	0.0110 (11)
N4	0.0248 (15)	0.0419 (16)	0.0570 (19)	−0.0042 (12)	0.0104 (15)	−0.0070 (13)
C1	0.0229 (15)	0.0400 (18)	0.045 (2)	0.0010 (13)	0.0164 (16)	0.0011 (14)
C2	0.0166 (13)	0.0316 (15)	0.0286 (16)	0.0007 (11)	0.0096 (13)	0.0033 (11)
C3	0.0210 (14)	0.0306 (15)	0.0285 (16)	0.0021 (11)	0.0144 (13)	0.0035 (12)
C4	0.0165 (13)	0.0294 (15)	0.0325 (16)	0.0005 (11)	0.0097 (13)	−0.0011 (12)
C5	0.0189 (13)	0.0267 (14)	0.0264 (15)	−0.0023 (11)	0.0106 (13)	0.0033 (11)
C6	0.0248 (15)	0.0264 (14)	0.0361 (17)	0.0032 (11)	0.0178 (14)	0.0025 (12)
C7	0.0316 (16)	0.0332 (15)	0.0286 (16)	−0.0008 (13)	0.0173 (15)	−0.0011 (12)
C8	0.0226 (15)	0.0326 (16)	0.0288 (16)	−0.0006 (12)	0.0110 (14)	0.0045 (12)
C9	0.0221 (14)	0.0280 (15)	0.0364 (17)	0.0031 (11)	0.0161 (14)	0.0044 (12)
C10	0.0232 (14)	0.0282 (15)	0.0302 (16)	−0.0013 (11)	0.0150 (14)	−0.0004 (11)
C11	0.040 (2)	0.055 (2)	0.0331 (19)	0.0070 (17)	0.0153 (18)	0.0064 (16)
C12	0.0237 (16)	0.0269 (15)	0.0414 (19)	−0.0029 (12)	0.0128 (15)	−0.0019 (13)

### Geometric parameters (Å, º)

**Table:**

Cl1—C6	1.724 (3)	N2—C4	1.327 (3)
Cl2—C10	1.723 (3)	N3—C3	1.340 (3)
S1—O1	1.479 (2)	N3—H3A	0.8800
S1—C2	1.739 (3)	N3—H3B	0.8800
S1—C1	1.844 (3)	N4—C12	1.144 (4)
F1—C1	1.329 (3)	C2—C3	1.398 (4)
F2—C1	1.325 (3)	C2—C4	1.408 (4)
F3—C1	1.321 (4)	C4—C12	1.437 (4)
F4—C11	1.344 (7)	C5—C10	1.388 (4)
F5—C11	1.281 (7)	C5—C6	1.389 (4)
F6—C11	1.331 (8)	C6—C7	1.379 (4)
F4'—C11	1.265 (9)	C7—C8	1.384 (4)
F5'—C11	1.357 (11)	C7—H7	0.9500
F6'—C11	1.253 (13)	C8—C9	1.373 (4)
N1—C3	1.359 (3)	C8—C11	1.500 (4)
N1—N2	1.379 (3)	C9—C10	1.387 (4)
N1—C5	1.418 (3)	C9—H9	0.9500
			
O1—S1—C2	108.69 (13)	C7—C6—C5	120.5 (3)
O1—S1—C1	102.58 (14)	C7—C6—Cl1	119.7 (2)
C2—S1—C1	96.30 (14)	C5—C6—Cl1	119.9 (2)
C3—N1—N2	113.4 (2)	C6—C7—C8	119.1 (3)
C3—N1—C5	125.6 (2)	C6—C7—H7	120.4
N2—N1—C5	119.7 (2)	C8—C7—H7	120.4
C4—N2—N1	103.1 (2)	C9—C8—C7	121.6 (3)
C3—N3—H3A	120.0	C9—C8—C11	119.3 (3)
C3—N3—H3B	120.0	C7—C8—C11	119.1 (3)
H3A—N3—H3B	120.0	C8—C9—C10	118.9 (3)
F3—C1—F2	108.4 (3)	C8—C9—H9	120.6
F3—C1—F1	107.4 (3)	C10—C9—H9	120.6
F2—C1—F1	107.9 (3)	C9—C10—C5	120.6 (3)
F3—C1—S1	110.0 (2)	C9—C10—Cl2	119.8 (2)
F2—C1—S1	110.0 (2)	C5—C10—Cl2	119.6 (2)
F1—C1—S1	112.9 (2)	F6'—C11—F4'	109.4 (8)
C3—C2—C4	104.7 (2)	F5—C11—F6	107.4 (6)
C3—C2—S1	127.2 (2)	F5—C11—F4	105.6 (5)
C4—C2—S1	128.1 (2)	F6—C11—F4	105.6 (5)
N3—C3—N1	122.4 (2)	F6'—C11—F5'	107.5 (9)
N3—C3—C2	132.0 (3)	F4'—C11—F5'	101.2 (7)
N1—C3—C2	105.5 (2)	F6'—C11—C8	113.4 (6)
N2—C4—C2	113.2 (2)	F4'—C11—C8	115.4 (5)
N2—C4—C12	119.1 (3)	F5—C11—C8	114.6 (4)
C2—C4—C12	127.8 (3)	F6—C11—C8	113.3 (4)
C10—C5—C6	119.3 (3)	F4—C11—C8	109.7 (4)
C10—C5—N1	121.4 (3)	F5'—C11—C8	108.9 (5)
C6—C5—N1	119.2 (2)	N4—C12—C4	179.7 (4)
			
C3—N1—N2—C4	1.3 (3)	N2—N1—C5—C6	−85.1 (3)
C5—N1—N2—C4	169.4 (2)	C10—C5—C6—C7	−0.2 (4)
O1—S1—C1—F3	67.2 (2)	N1—C5—C6—C7	−178.2 (2)
C2—S1—C1—F3	178.0 (2)	C10—C5—C6—Cl1	179.0 (2)
O1—S1—C1—F2	−173.4 (2)	N1—C5—C6—Cl1	1.0 (4)
C2—S1—C1—F2	−62.6 (2)	C5—C6—C7—C8	−0.3 (4)
O1—S1—C1—F1	−52.8 (2)	Cl1—C6—C7—C8	−179.5 (2)
C2—S1—C1—F1	58.0 (2)	C6—C7—C8—C9	−0.5 (4)
O1—S1—C2—C3	24.2 (3)	C6—C7—C8—C11	179.9 (3)
C1—S1—C2—C3	−81.4 (3)	C7—C8—C9—C10	1.8 (4)
O1—S1—C2—C4	−156.3 (3)	C11—C8—C9—C10	−178.7 (3)
C1—S1—C2—C4	98.1 (3)	C8—C9—C10—C5	−2.3 (4)
N2—N1—C3—N3	178.6 (3)	C8—C9—C10—Cl2	176.2 (2)
C5—N1—C3—N3	11.4 (4)	C6—C5—C10—C9	1.5 (4)
N2—N1—C3—C2	−0.3 (3)	N1—C5—C10—C9	179.5 (2)
C5—N1—C3—C2	−167.6 (3)	C6—C5—C10—Cl2	−177.0 (2)
C4—C2—C3—N3	−179.6 (3)	N1—C5—C10—Cl2	1.0 (4)
S1—C2—C3—N3	0.0 (5)	C9—C8—C11—F6'	164.8 (12)
C4—C2—C3—N1	−0.8 (3)	C7—C8—C11—F6'	−15.6 (12)
S1—C2—C3—N1	178.9 (2)	C9—C8—C11—F4'	37.5 (12)
N1—N2—C4—C2	−1.8 (3)	C7—C8—C11—F4'	−142.9 (11)
N1—N2—C4—C12	178.8 (3)	C9—C8—C11—F5	−38.1 (10)
C3—C2—C4—N2	1.7 (3)	C7—C8—C11—F5	141.4 (9)
S1—C2—C4—N2	−177.9 (2)	C9—C8—C11—F6	−161.9 (6)
C3—C2—C4—C12	−179.0 (3)	C7—C8—C11—F6	17.6 (6)
S1—C2—C4—C12	1.4 (5)	C9—C8—C11—F4	80.4 (6)
C3—N1—C5—C10	−96.6 (3)	C7—C8—C11—F4	−100.0 (6)
N2—N1—C5—C10	96.9 (3)	C9—C8—C11—F5'	−75.4 (7)
C3—N1—C5—C6	81.4 (3)	C7—C8—C11—F5'	104.1 (7)

### Hydrogen-bond geometry (Å, º)

**Table:**

*D*—H···*A*	*D*—H	H···*A*	*D*···*A*	*D*—H···*A*
N3—H3*A*···N4^i^	0.88	2.39	3.183 (3)	151
N3—H3*B*···O1^ii^	0.88	2.28	2.896 (3)	127
C9—H9···F2^i^	0.95	2.42	3.222 (3)	143

Symmetry codes: (i) *x*+1/2, −*y*+1/2, *z*+1/2; (ii) −*x*+2, *y*, −*z*+3/2.
